# Influence of ketamine, propofol or isoflurane on intraocular pressure, heart rate and blood pressure in healthy dogs premedicated with medetomidine and midazolam

**DOI:** 10.1002/vms3.1330

**Published:** 2023-11-27

**Authors:** Masoud Khaleghi, Ali Asghar Sarchahi, Hossein Kazemi Mehrjerdi, Mehdi Rasekh, Dariush Saadati

**Affiliations:** ^1^ Faculty of Veterinary Medicine University of Zabol Zabol Iran; ^2^ Faculty of Veterinary Medicine Department of Clinical Sciences Ferdowsi University of Mashhad Mashhad Iran; ^3^ Faculty of Veterinary Medicine Department of Clinical Sciences University of Zabol Zabol Iran; ^4^ Faculty of Veterinary Medicine Department of Food Hygiene University of Zabol Zabol Iran

**Keywords:** anaesthetics, blood pressure, cardiovascular variables, heart rate, intraocular pressure

## Abstract

**Background:**

According to the findings of several studies, sedatives and anaesthetics have different effects on the functioning of the cardiovascular system and intraocular pressure (IOP). For accurate diagnosis, treatment and surgery with minimal complications, it is necessary to be aware of the effects of sedatives and anaesthetics on the cardiovascular system and IOP.

**Objectives:**

The aim of this study was to evaluate the effects of sedatives (medetomidine and midazolam) and anaesthetics (ketamine, propofol and isoflurane) on IOP, heart rate (HR) and blood pressure in dogs.

**Methods:**

In this study, 10 dogs participated in three treatments using a randomised cross‐over design, with a 1‐week washout period between each treatment. Dogs in all treatments were premedicated with medetomidine and midazolam. Anaesthesia was induced using ketamine, propofol, or isoflurane and maintained for 60 min with the appropriate doses of each drug. The cardiovascular variables (heart rate, and systolic, diastolic and mean arterial pressures) and IOP were measured at different timepoints: before premedication (baseline values, T‐Bas), 15 min after medetomidine administration (T‐Med), 20 min after midazolam administration (T‐Mid) and at 15 (T‐15), 30 (T‐30), 45 (T‐45) and 60 (T‐60) min after anaesthesia induction.

**Results:**

Medetomidine significantly reduced the IOP and HR and did not significantly change the mean arterial pressure (MAP). Midazolam significantly reduced the IOP while did not significantly change the HR and MAP. Ketamine and isoflurane significantly increased the IOP and HR while did not significantly change the MAP. Propofol significantly increased the HR, but did not cause significant changes in IOP and MAP.

**Conclusions:**

Considering that anaesthetics are typically administered in conjunction with pre‐anaesthetic drugs, the increases in IOP induced by ketamine and isoflurane are not important, as the IOP did not exceed the baseline values. However, further studies are required to investigate these effects in patients with elevated IOP.

## Graphical abstract

The objective of this study was to evaluate the collective impact of pre‐anaesthetic (medetomidine and midazolam) and anaesthetic drugs (ketamine, propofol and isoflurane) on IOP, HR and blood pressure. Medetomidine and midazolam significantly reduced the IOP in dogs. Ketamine and isoflurane caused a significant increase in IOP in dogs. Considering that anaesthetics are typically administered with pre‐anaesthetic drugs, ketamine‐ and isoflurane‐induced increases in IOP are not important because the IOP did not exceed the values observed at baseline.

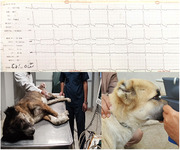

## 1 INTRODUCTION

General or local anaesthesia is one of the main components of surgery, used to eliminate pain and prevent patient reactions. Sometimes, due to non‐cooperation of animals, various types of sedatives or even general anaesthetics are used to calm restless and aggressive animals in order to facilitate their movement or to perform various diagnostic and therapeutic procedures (Vesal, 2015). Different pre‐anaesthetic and anaesthetic agents are used to induce anaesthesia in various animals. Some of the most commonly used drugs in this regard are medetomidine, midazolam, ketamine, propofol and isoflurane (Vesal, 2015).

Medetomidine, an alpha 2 adrenergic agonist, is a dose‐dependent sedative, analgesic and muscle relaxant in various animal species. The most important side effects of this drug include decreased heart rate (HR) and blood pressure, increased urination and the potential for vomiting in dogs (10%) and cats (over 50%) (Lemke, 2007; Pypendop & Verstegen, 1998). Medetomidine is commonly used as a pre‐anaesthetic before administering ketamine, sodium thiopental, propofol or inhalation anaesthesia (Bell et al., 2014).

Midazolam, as a benzodiazepine receptor agonist, facilitates the action of gamma‐aminobutyric acid and increases the influx of chloride ions into cells. Benzodiazepines reduce brain activity by decreasing the excitability of neurons (Hadley et al., 2012).

Ketamine is a rapid‐acting dissociative anaesthetic that acts on N‐methyl‐D‐aspartate (NMDA) receptors and induces anaesthesia by inhibiting them. This drug is typically used in conjunction with a muscle relaxant for diagnostic and surgical procedures (Zanos et al., 2018). Transient unwanted side effects, such as increased heart rate, high blood pressure, lacrimation, sneezing, excessive salivation, vomiting, muscle stiffness and excitement, have been reported after the administration of ketamine. However, there is still no consensus on neurological damage (Seliškar et al., 2007).

Propofol acts as a hypnotic in the central nervous system and is the most common anaesthetic used to induce and maintain anaesthesia. Propofol causes a decrease in cerebral blood flow and oxygen consumption, leading to reduced intracranial and mean arterial pressure (MAP) (Sahinovic et al., 2018). Induction of anaesthesia with propofol significantly increased IOP in dogs premedicated with dexmedetomidine‐hydromorphone and acepromazine‐hydromorphone (Hasiuk et al., 2014; Smith et al., 2019).

Isoflurane is a potent inhalation anaesthetic. It decreases the blood pressure by reducing vascular resistance. The muscle relaxation properties of isoflurane are more significant, and its impairing effect is less than that of halothane (Brosnan & Steffey, 2018). It typically does not affect heart rate unless the patient is allowed to become hypercapnic (Constantinides et al., 2011).

Most of the studies investigating the effects of these anaesthetics on intraocular pressure (IOP), heart rate (HR), and blood pressure have focused on the effect of either a single anaesthetic or a combination of two pre‐anaesthetic and anaesthetic drugs, such as midazolam‐ketamine, midazolam‐propofol and midazolam‐medetomidine (Ghaffari et al., 2010; Gunderson et al., 2013; Kojima et al., 1999; Verbruggen et al., 2000). However, to the best of our knowledge, the cumulative effects of pre‐anaesthetic medications and anaesthetics (as commonly administered in veterinary clinics) on IOP, HR and blood pressure have not been extensively investigated. Each of these drugs has its own advantages and disadvantages. For example, ketamine, propofol and isoflurane may increase IOP and HR, while the pre‐anaesthetic drugs used in this study may decrease them. Thus, we expect that the side effects of certain drugs will be covered by others, allowing for easy utilisation of these combinations in clinical settings. Therefore, the objective of this study was to evaluate the combined effects of pre‐anaesthetic and anaesthetic drugs (as routinely used in clinics) on IOP, HR and blood pressure. In the present study, we investigated the changes in IOP, HR and blood pressure following the administration of anaesthesia using three different protocols: ketamine, propofol and isoflurane, after premedication with medetomidine‐midazolam.

## 2 MATERIALS AND METHODS

This study was approved by the Research Ethics Committee of the University of Zabol, Zabol, Iran (Approval ID: IR.UOZ.REC.1401.005). This study was conducted on ten adult intact mixed breed dogs, consisting of four males and six females. The dogs had an average age of 24.4 ± 6.6 months (ranging from 16 to 36 months) and an average weight of 22.2 ± 4.6 kg (ranging from 13 to 28 kg). All dogs included in the study were clinically healthy.

Two weeks before the start of the experiment, the dogs were kept under constant conditions in the kennel of the Teaching Hospital of the Faculty of Veterinary Medicine, Ferdowsi University of Mashhad, Mashhad, Iran. Routine physical, cardiovascular, and ophthalmic examinations were conducted, which included auscultation of the heart and lungs, ECG recording, blood pressure measurement (systolic, diastolic and mean arterial pressure), observation of the globe and eyelids, vision detection (by menace response), pupillary light reflex (PLR) testing (both direct and consensual), examination with a direct and panoptic ophthalmoscope, fluorescein staining of the cornea and tonometry (using a rebound tonometer). These examinations revealed no abnormality. Therefore, only dogs without any ophthalmological and cardiovascular diseases were included in the study. In addition, dogs with intraocular pressure less than 5 or greater than 25 mmHg, or with an IOP difference of more than 4 mmHg between the right and left eye were excluded from the study. All dogs were habituated to the experimental room and fasted for 12 h before treatment and investigated in the same environment under the same light conditions.

All 10 dogs participated in each of the 3 treatments in a cross‐over design, with at least a 1‐week washout period between them. The dogs were randomly assigned to each treatment group. In other words, each dog was assigned a number and in a systematic random sampling, each dog was first randomly placed in one of the ketamine, propofol, or isoflurane groups. After a 1‐week washout period from the previous experiment, they were then randomised into one of the two other groups.

In each dog, an IV catheter was placed in the cephalic vein, and Ringer's solution was administered at a rate of 10 mL/kg/h. Vital signs of dogs, including heart rate (via ECG), respiratory rate (via auscultation), and rectal temperature (with a medical thermometer), were measured at all timepoints. In all treatments, the desired variables, including IOP, ECG, systolic, diastolic and MAP were measured and recorded as baseline values (T‐Bas). Then, for sedation, medetomidine (Dorbene 1 mg/mL, Syva Laboratories S.A., Leon, Spain) was injected intramuscularly into the hamstring muscles at a dose of 15 μg/kg (Lemke, 2007), and the desired variables were measured after 15 min (T‐Med). Then, to complete the pre‐anaesthesia, midazolam (Midazolex, Exir Pharmaceutical Company, Boroujerd, Iran) was injected intramuscularly into the hamstring muscles at a dose of 0.25 mg/kg (Kropf & Hughes, 2018) and the desired variables were measured after 20 min (T‐Mid). Then, in the first treatment, ketamine hydrochloride (Ketamin 10%, Bremer Pharma GmbH, Warburg, Germany) was intravenously injected at a dose of 6 mg/kg (within 60 s) for induction and at a dose of 3 mg/kg once every 15 min for the continuation of anaesthesia (Hellebrekers et al., 1998). In the second treatment, propofol (Lipuro 10 mg/mL, B. Braun Melsungen AG, Melsungen, Germany) was administered intravenously at a dose of 6.5 mg/kg (within 60 s) for induction and a dose of 0.25 mg/kg/min for maintenance of anaesthesia (Berry, 2015). The maintenance doses of propofol were administered using a syringe infusion pump (SP‐510, JMS Co. LTD, Hiroshima, Japan). In the third treatment, anaesthesia was initially induced with 5% isoflurane (Piramal Critical Care LTD, West Drayton, UK) in 100% oxygen through a mask. Tracheal intubation was then immediately performed and connected to the anaesthesia machine (Piramal Critical Care LTD, West Drayton, UK). When the desired anaesthesia was induced, it was maintained with 2% isoflurane in 100% oxygen (Mutoh et al., 1997). An anaesthetic machine with a rebreathing circuit method was used. This machine adjusted the concentration of isoflurane in the vaporiser while the dogs were breathing spontaneously. The anaesthesia was maintained for 60 min with the appropriate maintenance doses for each drug. During the anaesthesia, the variables were measured every 15 min, i.e., at 15 (T‐15), 30 (T‐30), 45 (T‐45) and 60 (T‐60) min after the induction of anaesthesia. Only the dogs in the isoflurane group were intubated and all the dogs in three groups were breathing spontaneously.

### 2.1 Intraocular pressure measurement

The intraocular pressure was measured while the animals were in the sternal position using a rebound tonometer (TA01i Tonometer, iCare, Finland). In all dogs, according to the manufacturer's recommendation, the tonometer was placed perpendicular to the cornea at a distance of 4–8 mm and the same conditions were applied. All measurements were performed between 12:00 PM and 3:00 PM to minimise the effect of diurnal IOP variation on the experiment. In order to conduct the study in a blinded manner, all measurements were taken by the same examiner (AAS) who was unaware of the drugs being used. At each timepoint, IOP was measured three times, and the average was recorded.

### 2.2 Blood pressure measurement

To measure the blood pressure, the dogs were placed in right lateral recumbency and non‐invasive blood pressure, including MAP, systolic arterial pressure (SAP), and diastolic arterial pressure (DAP) was measured by oscillometry using a cardiopulmonary monitoring device (Cardioset ARAD P10, Sairan Electro Optics Industries Co., Esfahan, Iran) and a suitable cuff (a cuff with a width of 40% of the limb circumference) that was placed on the antebrachium of the left thoracic limb.

### 2.3 Recording the ECG and measuring the heart rate

The ECG was recorded with the animal in right lateral recumbency by connecting electrodes to each limb using an electrocardiograph device (Bionics, BCM‐600, Gangwon‐do, South Korea). Bipolar and unipolar leads (I, II, III, aVR, aVL, aVF) were used to record the ECG, and the heart rate was determined by counting the number of beats on the ECG.

### 2.4 Statistical analysis

Data were analysed using SPSS 25.0 software for Windows and Graphpad Prism version 9. All values are reported as means ± SE. The variables measured in the dogs of all three groups (30 measurements) at the timepoints before anaesthetic injections (i.e., T‐Bas, T‐Med and T‐Mid) were pooled and were compared using the Repeated Measures ANOVA and Dunnett's supplementary test. In addition, the variables measured at the timepoints after anaesthesia induction were compared using the Repeated Measures ANOVA and Dunnett's supplementary test. A value of *p* < 0.05 was considered statistically significant.

## 3 RESULTS

Table 1 displays the measured variables at different timepoints: before the drug injections (T‐Bas), after medetomidine (T‐Med) and after midazolam (T‐Mid) injections. As shown in this table, after medetomidine injection, the IOP of both eyes (*p* < 0.05) and HR (*p* < 0.01) significantly decreased compared to the baseline values. However, there were no significant changes in SAP, DAP and MAP (*p* > 0.05).

**TABLE 1:** Mean and standard error (mean ± SE) of measured variables of dogs (30 measurements) at different timepoints: before drug injections (T‐Bas), after medetomidine (15 μg/kg IM) (T‐Med) and after midazolam (0.25 mg/kg IM) (T‐Mid) injections.

variable	T‐Bas	T‐Med	T‐Mid
IOP_L_ (mmHg)	11.3 ± 0.7	9.2 ± 0.6^**^	7.8 ± 0.3^**^
IOP_R_ (mmHg)	11.1 ± 0.6	10 ± 0.6^*^	8.6 ± 0.4^**^
SAP (mmHg)	119 ± 5.4	115 ± 6.1	120 ± 5.3
DAP (mmHg)	81 ± 3.4	86 ± 4.5	84 ± 4
MAP (mmHg)	95 ± 4.1	97 ± 4.7	96 ± 4.4
HR (beats/min)	106 ± 5.3	51 ± 3.4^**^	49 ± 2.6

*Note*: T‐Med data are compared with T‐Bas and T‐Mid data are compared with T‐Med.

Abbreviations: IOP_L_, intraocular pressure in left eyes; IOP_R_, intraocular pressure in right eyes; SAP, systolic arterial pressure; DAP, diastolic arterial pressure; MAP, mean arterial pressure; HR, heart rate; T‐Bas, baseline value; T‐Med, 15 min after medetomidine injection; T‐Mid, 20 min after midazolam injection.

* Significant difference at the level of *p* < 0.05.

** Significant difference at the level of *p* < 0.01.

After the injection of midazolam, the IOP in both eyes decreased significantly compared to the injection of medetomidine (*p* < 0.01). Other variables did not change significantly (*p* > 0.05).

Table 2 displays the measured variables from the time of midazolam injection until 60 min later for three treatment groups: ketamine, isoflurane and propofol. For each variable, the timepoint of midazolam injection (T‐Mid) was considered as the reference value, and the values of the variables at the subsequent timepoints were compared to it.

**TABLE 2:** Mean and standard error (mean ± SE) of measured variables in ten dogs at different timepoints after administration of anaesthetic drugs.

variable	Treatment	T‐Mid	T‐15	T‐30	T‐45	T‐60
IOP_L_ (mmHg)	Ketamine	7.9 ± 0.6	8.4 ± 0.5	8.4 ± 0.5	7.9 ± 0.5	7.5 ± 0.5
	Propofol	7.9 ± 0.6	11.3 ± 2.0	10.3 ± 1.3	9.4 ± 1.6	8.4 ± 0.9
	Isoflurane	7.4 ± 0.4	10.5 ± 1.0	10.9 ± 0.6^**^	9.8 ± 0.6	9 ± 0.9
IOP_R_ (mmHg)	Ketamine	8.9 ± 0.8	10.4 ± 0.9	9.8 ± 0.6^*^	9.5 ± 0.7	8.7 ± 0.4
	Propofol	8.1 ± 0.6	12.1 ± 1.7	12 ± 1.3	10.1 ± 1.2	10.9 ± 1.0
	Isoflurane	8.9 ± 0.4	10.4 ± 0.6	12.2 ± 0.9^*^	11.9 ± 0.7^*^	10 ± 0.9
SAP (mmHg)	Ketamine	104 ± 9	113 ± 9	126 ± 7	124 ± 8	119 ± 8
	Propofol	130 ± 8	134 ± 7	135 ± 5	126 ± 5	133 ± 4
	Isoflurane	125 ± 9	120 ± 3	123 ± 4	127 ± 6	119 ± 6
DAP (mmHg)	Ketamine	70 ± 6	80 ± 9	94 ± 7	89 ± 9	92 ± 7
	Propofol	94 ± 6	96 ± 4	90 ± 3	85 ± 4	95 ± 3
	Isoflurane	86 ± 8	71 ± 4	71 ± 6	65 ± 4	67 ± 5
MAP (mmHg)	Ketamine	80 ± 7	91 ± 9	104 ± 7	101 ± 8	100 ± 7
	Propofol	106 ± 6	108 ± 4	107 ± 4	101 ± 4	108 ± 3
	Isoflurane	100 ± 8	88 ± 3	88 ± 5	83 ± 4	86 ± 5
HR (beats/min)	Ketamine	43 ± 3	69 ± 6^**^	70 ± 6^**^	72 ± 7^**^	78 ± 6^**^
	Propofol	52 ± 5	77 ± 8^**^	67 ± 6	65 ± 8	69 ± 6
	Isoflurane	52 ± 5	92 ± 6^**^	88 ± 8^*^	88 ± 7^*^	84 ± 5^**^

*Note*: The measured variables after the induction of anaesthesia (T‐15, T‐30, T‐45 and T‐60) were compared with the timepoint of midazolam injection (T‐Mid).

Abbreviations: IOP_L_, intraocular pressure in left eyes; IOP_R_, intraocular pressure in right eyes; SAP, systolic arterial pressure; DAP, diastolic arterial pressure; MAP, mean arterial pressure; HR, heart rate; T‐Mid, 20 min after midazolam injection; T‐15, T‐30, T‐45 and T‐60, timepoints of measuring the variables at 15, 30, 45 and 60 min after induction of anaesthesia in the treatment groups.

* Significant difference at the level of *p* < 0.05.

** Significant difference at the level of *p* < 0.01.

In ketamine treatment, the IOP gradually increased in both eyes so that at 30 min, the IOP of the right eye was significantly higher than the IOP at the time of midazolam injection (*p* < 0.05). However, IOP changes in the left eye were not significant at any timepoint compared to the midazolam timepoint. Then it gradually decreased, so that in the 60th minute, the IOP of both eyes was not significantly different from the value measured after midazolam injection. The heart rate significantly increased at 15, 30, 45 and 60 min after the induction of anaesthesia compared to the value measured after midazolam injection (*p* < 0.01).

In propofol treatment, the IOP of both eyes increased in the 15th minute and then began to decrease until the 60th minute. These changes were not statistically significant compared to the value measured after the injection of midazolam. On the other hand, the HR increased significantly in the 15th minute (*p* < 0.01). It then gradually decreased during the continuation of anaesthesia, so that no significant difference was observed at the subsequent timepoints compared to the value measured after midazolam injection.

In the isoflurane treatment, the IOP of both eyes began to rise and reached its maximum after 30 min, so that at this timepoint, the IOP was significantly higher than the value measured after the injection of midazolam (*p* < 0.05). Then, it started to decrease so that the IOP of both eyes at 60 min was not significantly different from the value measured after midazolam injection (*p* > 0.05). The HR increased significantly in the 15th minute (*p* < 0.01). However during the course of anaesthesia, it gradually decreased until the 60th minute, but it never returned to the initial value and was remained significantly higher than the value measured after midazolam injection at all timepoints (*p* < 0.05).

The comparison of measured variables between the three treatments is given in Table 3. As shown in this table, DAP and MAP, were the lowest in the isoflurane treatment and the highest in the propofol treatment. These differences were significant in the isoflurane treatment compared to the ketamine and propofol treatments (*p* < 0.001). The HR was the lowest in the propofol treatment and the highest in the isoflurane treatment, and this difference was significant (*p* = 0.045). The HR in the ketamine treatment was not significantly different from the other two treatments. However, it was significantly lower in the propofol treatment compared to the isoflurane treatment.

**TABLE 3:** Comparison of measured variables among three groups (ketamine/ propofol/ isoflurane).

Variable	Ketamine	Propofol	Isoflurane	*p* Value
IOP_L_ (mmHg)	8.1 ± 0.7^a^	10 ± 0.8^a^	9.8 ± 0.8^a^	0.155
IOP_R_ (mmHg)	9.6 ± 0.6^a^	11.6 ± 0.8^a^	11.1 ± 0.7^a^	0.199
SAP (mmHg)	121 ± 5^a^	132 ± 4^a^	121 ± 4^a^	0.101
DAP (mmHg)	88 ± 5^a^	91 ± 4^a^	68 ± 3^b^	<0.001
MAP (mmHg)	99 ± 4^ab^	105 ± 4^b^	85 ± 3^a^	0.002
HR (beats/min)	72 ± 6^ab^	67 ± 6^b^	90 ± 6^a^	0.045

*Note*: Different letters in each row indicate statistically significant differences. The values shown in each treatment group represent the average values (mean ± SE) obtained at 15, 30, 45 and 60 timepoints. The *p* value in this table is related to the between‐subjects effects in repeated measures.

Abbreviations: IOP_L_, intraocular pressure in left eyes; IOP_R_, intraocular pressure in right eyes; SAP, systolic arterial pressure; DAP, diastolic arterial pressure; MAP, mean arterial pressure; HR, heart rate.

## 4 DISCUSSIONS

In the present study, the intramuscular administration of medetomidine produced profound sedation in all dogs. In addition, the IOP of the left and right eyes decreased significantly (*p* < 0.05). This reducing effect of medetomidine on IOP is consistent with the findings of other studies (Kanda et al., 2015; Rauser et al., 2012; Verbruggen et al., 2000). However, none of these studies have mentioned the exact mechanism by which medetomidine reduces IOP. Kanda et al. (2015) hypothesised three possible mechanisms for the reduction of IOP after the injection of alpha‐2 agonists: (1) activation of alpha‐2 adrenergic prejunctional receptors inhibits the release of norepinephrine, leading to a reduction in the production of aqueous humour; (2) vasoconstriction of the ciliary body leads to a decrease in blood flow within the ciliary body; (3) activation of alpha‐2 adrenergic epithelial receptors inhibits adenylyl cyclase, which reduces the production of aqueous humour (Kanda et al., 2015).

In the present study, HR significantly decreased after medetomidine injection (*p* < 0.01). Alpha‐2 agonists cause vasoconstriction by directly acting on alpha‐2 receptors in the smooth muscles of the vascular wall, thereby increasing vascular resistance and blood pressure. An increase in blood pressure leads to a decrease in HR (Scheinin et al., 1987; Vainio & Palmu, 1989). Alpha‐2 agonists also increase vagal nerve tone and decrease sympathetic nerve activity by affecting central alpha‐2 receptors, resulting in a decrease in HR (Murrell & Hellebrekers, 2005; Vainio & Palmu, 1989).

In the present study, the IOP of both eyes significantly decreased after midazolam injection (*p* < 0.01). The effect of midazolam on IOP has been reported to be contradictory in different studies. In some studies, administration of midazolam blunted or even reduced propofol‐induced increases in IOP (Webb et al., 2018), while in other studies, no change was observed (Ghaffari et al., 2010). Therefore, the mechanism of action of midazolam on IOP is still unclear. In accordance with the present results, previous studies have demonstrated that midazolam does not significantly change the MAP and HR in dogs (Kropf & Hughes, 2018). Nevertheless, when an alpha‐2 agonist (medetomidine) is used in combination with a muscle relaxant (midazolam), they have a synergistic effect, causing bradycardia and atrioventricular block, and significantly reduce central noradrenergic neurotransmitters (Hayashi et al., 1995; Kojima et al., 1999). In contrast, the injection of midazolam alone only has a weak sedative effect. However, in the present study, some of the effects of midazolam may have been masked due to the prior administration of medetomidine.

One of the reasons for the increase in IOP after ketamine injection in the present study could be the increase in extraocular muscle tone. However, the mechanism and mode of action of ketamine on these muscles are not clear (Ghaffari et al., 2010; Kovalcuka et al., 2013). Hofmeister et al. (2006) observed that during deep anaesthesia with ketamine (10 mg/kg), the tone of the muscles, particularly the extraocular muscles, does not increase. As a result, the IOP remains unchanged. However, in low‐dose anaesthesia with ketamine (5 mg/kg IV), the IOP increases (Hofmeister et al., 2006). The dose used in the present study was lower than that in other studies, and this could be one of the reasons for the observed differences.

In the present study, the HR began to increase after the administration of ketamine and showed a continuous upward trend until 60 min after induction. This increase was significantly different from the HR measured after the injection of midazolam at all timepoints (*p* < 0.01). On the other hand, in this study, similar to other studies, there was no significant change in MAP after ketamine injection. Therefore, the increase in HR is likely due to the effect of ketamine on the sympathetic nerves, which leads to an increase in the concentration of catecholamines in the nerve terminals. This characteristic of ketamine, which increases the concentration of catecholamines, neutralises the negative inotropic effect of ketamine (Wiryana et al., 2017).

In the present study, after the induction of anaesthesia with propofol, the changes in IOP at different timepoints were not significantly different from the midazolam timepoint. It has been reported that the increase in IOP after propofol injection is caused by an increase of CO2 levels and an increase in the activity of the carbonic anhydrase enzyme. Additionally, there is evidence of a central nervous mechanism that independently affects the production of aqueous humour, regardless of arterial blood pressure (Batista et al., 2000; Hofmeister et al., 2009; Hasiuk et al., 2014). Webb et al. (2018) reported that propofol increases IOP in non‐premedicated dogs without glaucoma. Additionally, the administration of pre‐anaesthetic drugs reduces the propofol‐induced elevation of IOP (Webb et al., 2018). In the present study, the administration of pre‐anaesthetics may have prevented the further increases in IOP after propofol administration. The significant increase in heart rate observed after propofol administration in the present study is consistent with previous reports (Cattai et al., 2018; Mayer et al., 1993; Wouters et al., 1995). The increase in HR caused by propofol may be attributed to the drug's inhibition of sympathetic tone and its ability to reduce peripheral vascular resistance, leading to vasodilation. This can stimulate baroreceptors and result in an elevated heart rate as a compensatory mechanism to maintain cardiac output (Su et al., 2022).

As stated in the results section, isoflurane caused significant changes in IOP. Chae et al. (2021) reported that isoflurane increases IOP in rabbits (Chae et al., 2021). On the other hand, Kılıc and Unsaldı (2009) reported that the induction of anaesthesia with isoflurane after the injection of xylazine reduces IOP in dogs. The increase in IOP observed in the present study is contrary to the study of Kilic and Unsaldi (2009) in dogs and consistent with the study of Chae et al. (2021) in rabbits. Investigating the cause of IOP increase is beyond the scope of this study; however it has been reported that certain inhaled anaesthetics can cause an increase in IOP due to elevated central venous pressure. We speculate that a similar effect may have occurred with isoflurane in the present study (Chae et al., 2021; Schreuder & Linssen, 1972).

Marano et al. (1996) reported that isoflurane affects the heart in rabbits by reducing the tone of the vagus nerves. This reduction in tone allows the remaining sympathetic tone to predominate over the parasympathetic tone, resulting in an increase in HR (Marano et al., 1996). It is assumed that in the present study, the injection of medetomidine decreased sympathetic nerve tone and led to a decrease in the HR. Subsequently, the induction of anaesthesia with isoflurane also decreased parasympathetic nerve tone, resulting in a predominance of the sympathetic nerve tone over the parasympathetic nerve tone and an increase in heart rate.

One of the limitations of the present study was the small number of dogs used in each group, which may have an adverse effect on the statistical results. To reduce the adverse effect of this limitation, we tried to select dogs with similar age and weight in order to minimise statistical dispersion.

Another limitation of the present study was the change in the animal's position from lateral to sternal in order to measure intraocular pressure, which could potentially result in alterations in IOP values. The best‐recommended position for measuring IOP by a portable rebound tonometer is the sternal position. We tried to have a short pause after changing the animals to a sternal position to allow them to fully relax before measuring the IOP. In addition, when administering propofol and isoflurane, it was challenging to measure the IOP due to the downward movement of the eyes. At times, we had to delicately retract the third eyelid to obtain accurate measurements. Since the order of measurement between right and left eyes may affect IOP (Pekmezci et al., 2011), we always measured the IOP first in the left eye and then in the right eye.

In the present study, due to the close proximity of the timepoints, blood pressure was measured only once at each timepoint. However, it would have been preferable to measure blood pressure at least three times at each timepoint and calculate the average. This reduces the accuracy of the measured values (third limitation). However, to enhance the accuracy of the measurements, we utilised an advanced monitoring device that is commonly employed in human hospitals.

The fourth limitation of the present study was the separate administration of medetomidine and midazolam as pre‐anaesthetic drugs. Premedication drugs are typically administered concurrently, but we wanted to investigate the individual effects of each drug. In our study, we have investigated the effects of medetomidine exclusively and the effects observed after the administration of midazolam can be partially attributed to this drug (midazolam). If we mixed them, its effect would be masked by midazolam, and the effects of both drugs would not be clearly determined.

The fifth limitation of the present study was the lack of SpO2 and ETCO2 monitoring. This can affect the blood pressure and HR if significantly increased. We wanted to check the normal conditions. In many minor surgeries, animals are operated without the need for oxygen and only with natural breathing. Our goal here was to investigate the parameters under natural conditions in a light surgical procedure that occurs many times.

In the present study, ketamine was administered as a bolus every 15 min. This may cause fluctuations in plasma ketamine concentration and may affect IOP measurement (Hofmeister et al., 2006). However, in this study, by repeatedly injecting ketamine every 15 min and evaluating the deep pain in the limbs, we ensured that the depth of anaesthesia remained at an acceptable level to avoid any negative effect on various parameters, particularly IOP.

In the present study, we considered the measurement times based on the peak effect time of the drugs (Ahmad et al., 2011; Kuusela et al., 2000). Therefore, we measured the parameters 15 and 20 min after administration of medetomidine and midazolam, respectively. In the case of anaesthetic drugs, we aimed to maintain a consistent and appropriate level of anaesthesia depth, and we monitored the variables every 15 min. With these measurement intervals, some data, particularly cardiovascular data, may be lost. However, we made an effort to conduct the measurements at the most optimal times in order to minimise data loss and obtain the most accurate results. Therefore, if there were any changes in the parameters during this period, they would definitely be evident.

The present study was conducted using a specific protocol, so its results are specific to this protocol. The results may vary with different pre‐anaesthesia and anaesthesia combination methods. This study was conducted on dogs that were clinically healthy. Therefore, these results may differ in dogs suffering from cardiovascular or ocular diseases.

## 5 CONCLUSION

The results of the present study showed that pre‐anaesthetic drugs, medetomidine and midazolam, at the doses studied, led to a significant decrease in IOP and HR (*p* < 0.05). On the contrary, ketamine and isoflurane increased IOP and HR, and propofol increased HR, particularly within the first few minutes of administration. Considering that anaesthetic drugs are typically administered together with pre‐anaesthetics, there is no specific concern for an increase in IOP and HR after the use of ketamine, propofol and isoflurane when used simultaneously with medetomidine or midazolam. This is because the IOP value after the administration of anaesthetic drugs still did not reach the initial normal value before the administration of pre‐anaesthetic drugs. However, this conclusion may not be true in patients with elevated IOP, and further studies are needed to validate this finding.

## AUTHOR CONTRIBUTIONS

Conceptualisation: Ali Asghar Sarchahi. Methodology: Ali Asghar Sarchahi, Masoud Khaleghi, Hossein Kazemi Mehrjerdi, Mehdi Rasekh, Dariush Saadati. Writing – original draft preparation: Ali Asghar Sarchahi. Writing – review and editing: Ali Asghar Sarchahi, Masoud Khaleghi, Hossein Kazemi Mehrjerdi, Mehdi Rasekh, Dariush Saadati. Supervision: Ali Asghar Sarchahi.

## CONFLICT OF INTEREST STATEMENT

The authors declare no conflicts of interest.

## ETHICS STATEMENT

The study protocol was approved by the Research Ethics Committee of the University of Zabol, Iran (Approval ID: IR.UOZ.REC.1401.005).

## Data Availability

All relevant data are in the manuscript. The datasets used and analysed in this study are available from the corresponding author upon reasonable request.
